# A simple route to vertical array of quasi-1D ZnO nanofilms on FTO surfaces: 1D-crystal growth of nanoseeds under ammonia-assisted hydrolysis process

**DOI:** 10.1186/1556-276X-6-564

**Published:** 2011-10-25

**Authors:** Akrajas Ali Umar, Mohd Yusri Abd Rahman, Rika Taslim, Muhamad Mat Salleh, Munetaka Oyama

**Affiliations:** 1 Institute of Microengineering and Nanoelectronics (IMEN), Universiti Kebangsaan Malaysia, 43600, Bangi, Selangor, Malaysia; 2 College of Engineering, Universiti Tenaga Nasional, 43000, Kajang, Selangor, Malaysia; 3 Department of Materials Chemistry, Graduate School of Engineering, Kyoto University, Nishikyo-ku, Kyoto 615-8520 Japan

**Keywords:** ZnO quasi-NRs, nanofilms, vertical array, hydrolysis process, seed-mediated method

## Abstract

A simple method for the synthesis of ZnO nanofilms composed of vertical array of quasi-1D ZnO nanostructures (quasi-NRs) on the surface was demonstrated via a 1D crystal growth of the attached nanoseeds under a rapid hydrolysis process of zinc salts in the presence of ammonia at room temperature. In a typical procedure, by simply controlling the concentration of zinc acetate and ammonia in the reaction, a high density of vertically oriented nanorod-like morphology could be successfully obtained in a relatively short growth period (approximately 4 to 5 min) and at a room-temperature process. The average diameter and the length of the nanostructures are approximately 30 and 110 nm, respectively. The as-prepared quasi-NRs products were pure ZnO phase in nature without the presence of any zinc complexes as confirmed by the XRD characterisation. Room-temperature optical absorption spectroscopy exhibits the presence of two separate excitonic characters inferring that the as-prepared ZnO quasi-NRs are high-crystallinity properties in nature. The mechanism of growth for the ZnO quasi-NRs will be proposed. Due to their simplicity, the method should become a potential alternative for a rapid and cost-effective preparation of high-quality ZnO quasi-NRs nanofilms for use in photovoltaic or photocatalytics applications.

**PACS**: 81.07.Bc; 81.16.-c; 81.07.Gf.

## Introduction

ZnO nanocrystals, such as nanorods, nanowires and nanoparticles, have been receiving a growing research attention in the last few decades due to their unique electrical and optical properties [1-6]. ZnO is characterised by a wide direct band gap of 3.37 eV that indicates the potential use in blue light-emitting [7] devices application. Their high electron mobility (bulk ZnO 150 to 350 cm^2 ^V^-1 ^s^-1^), high exciton binding energy (60 meV) and long diffusion length [8] make them great material candidates for electronics [9], optoelectronics [10,11] devices and solar cell and photocatalyst applications [12-14]. The synthesis of ZnO in the form of nanorods or nanowires is expected to further enhance their intrinsic property as the results of quantum effect.

Many approaches have been demonstrated for the preparation of ZnO nanorods and nanowires on solid substrate so far. They include, but are not limited to, vapour-liquid-solid (VLS) [15], metal organic vapour phase epitaxy [16,17], plasma-enhanced chemical vapour deposition [18,19] and a simple vapour-solid process [20]. Amongst the available techniques, a vapour-liquid-solid (VLS) has been recognised as a versatile method to prepare high-quality ZnO oxide nanorods. The detail of the process and the promising properties of ZnO nanostructures prepared using these methods have also been well summarised in [1-6]. Although high-quality ZnO nanorods and nanowires can be successfully realised, such as controlled structures, growth orientation and properties, these techniques are recognised to comprise several major drawbacks, such as high-temperature process (typically approximately 1,000°C) to facilitate liquidifying and evaporating the zinc precursor and the growth. In addition, since usual procedure requires metal catalysts to promote and direct the ZnO nanorods growth, the ZnO product certainly is seriously contaminated by them. In many applications, this is definitely unexpected since they may superimpose the intrinsic properties of the ZnO itself. Thus, the unique properties of ZnO nanorods could not be harvested. After growth effort to remove them has also been demonstrated, but has come up with limited success. Due to the unique properties of ZnO nanorods and their potential function in currently existing applications, a low-temperature process and catalyst-free growth for nanorods on the surface should be continuously demonstrated.

So far, well known and widely used techniques of catalyst-free and low-temperature growth process for 1D ZnO nanostructures on the surface are represented by anodic aluminium oxide (AAO) template electrochemical [21] and hydrothermal [22-24] methods. For the case of the AAO template method, high-quality vertical array tubular ZnO nanostructures on the surface have been normally realised at a room-temperature processing. However, despite the fact that after growth templates removal indicates a diminutive problem and effect on the grown-up nanostructures, this method shows a strict limitation on the reducing of the nanorods or nanotubes diameter as an inadequacy in controlling the dimension of the AAO template itself. A hydrothermal method seems to be the potential approach for a better synthetic control for a catalyst-free 1D ZnO growth on the substrate surface. This technique realises the growth of vertically oriented ZnO nanorods on the surface from the nanoseeds under a low-temperature hydrothermal process (approximately 60°C to 150°C) in an autoclave. Typical growth time is approximately 4 to 12 h. Highly ordered ZnO nanorods on the surface have been produced by coupling with a lithography seeding process [25]. Improved results could be likely further obtained via coupling with a sonochemical [26] or microwave-assisted [27] hydrothermal process. In contrast to such interesting properties, however, hydrothermal techniques actually impose a tight control over the preparation process, such as temperatures and atmosphere (normally using autoclave), to obtain preferred ZnO products. Also, in the growth process, this technique is relatively time-consuming (typical time for projecting 50-nm nanorods is approximately >4 h) so that the preparation of ZnO nanorods with high aspect ratio is a challenging process. In addition, since the nature of this technique produces ZnO product not only on the target surface but also throughout the container, it requires an appropriate position of the target surface for obtaining a desired ZnO nanorods structure, inferring that it is a complex procedure. Therefore, considering the broad spectrum of ZnO nanorods applications, the preparation of ZnO nanorods with a simple and rapid process is highly demanded.

Here, we demonstrate an alternative method for preparing high-density, vertically oriented quasi-1D ZnO nanofilms on the surfaces via a 1D crystal growth of nanoseeds under a simple ambient-temperature hydrolysis process of zinc salt in the presence of ammonia with a relatively short growth period. In a typical process, the growth time to project the nanoseed into quasi-NRs morphology was approximately 3 min and this can produce quasi-NRs with a final length of up to approximately 150 nm. The morphology of the quasi-NRs was noticed to depend on the concentration of the ammonia and the zinc precursor in the reaction. X-ray diffraction (XRD) characterisation on the as-prepared sample surprisingly discovered that the samples had a phase purity of ZnO without the presence of any zinc complexes. A room-temperature optical absorption spectroscopy analysis surprisingly revealed that the nanostructures were high-degree crystallinity in nature, which was indicated by the presence of two distinct excitonic characters, namely *A*- and *B*-excitons, on the spectrum. Although better shape control is not yet achieved in the present report, due to the simplicity of the process, the present method should become a potential approach for the preparation of vertically oriented quasi-NRs ZnO nanofilms on the surface for use in currently existing applications.

## Experimental

Quasi-1D ZnO nanostructures on FTO (Solartron, Oak Ridge, TN, USA) surface were prepared via 1D crystal growth of nanoseeds on the surface in the presence of ammonia, adopting our previous approach in preparing CuO nanowires on the surface [28]. This method consists of two steps, namely seeding and growth processes. The following are typical procedures for the preparation of ZnO quasi-NRs on the FTO surface.

### Seeding process

ZnO nanoseeds on the FTO surface were prepared using an alcohothermal seeding method. In the typical process, a thin layer of ethanoloic solution of zinc acetate dihydrate (Zn (CH_3_COO)_2 _2H_2_O, Across) on a clean FTO surface was firstly prepared using a two-step spin-coating process at 400 and 2,000 rpm for 6 and 30 s, respectively. The concentration of Zn (CH_3_COO)_2 _2H_2_O used was 0.01 M. The sample was then dried up at 100°C on a hot-plate for 15 min. This procedure was repeated three times. After that, the sample was annealed in air at 350°C for 1 h. This process may produce high-density ZnO nanoseeds with sizes ranging from 5 to 10 nm on the surface.

### Growth process

The ZnO quasi-NRs were grown from the attached nanoseeds by simply immersing the nanoseeds-attached FTO into a 35-ml glass vial containing 10 mL of 10 mM aqueous solution of zinc acetate dihydrate (Zn (CH_3_COO)_2 _2H_2_O, Aldrich Chemical Co., Milwaukee, WI, USA). The sample was kept in a vertical position in the vial during the reaction by hanging it using adhesive tape. The solution was then mildly stirred during the reaction using a 10-mm magnetic stirrer bar. After that, a 30 μL of 30% ammonia solution (NH_3_, Aldrich) was added drop wisely into the reaction using a micropipette. This composition is referred as standard reaction later. The time interval for the additions of NH_3 _drops was approximately 1 min. The clear solution of zinc acetate immediately changed to a translucent bluish colour for the first 1 to 3 min of the process (inferring a rapid hydrolysis of zinc complexes in the growth solution) and then disappeared, a reflection of complete olation process of zinc complexes on the nanoseeds surface. This phenomenon was again obtained every time the ammonia was added into the solution. A tiny whitish suspension was sometimes observed if the reaction time was extended or a high concentration of ammonia was used. The reaction was allowed to continue for up to 5 min for a growth process. The effect of ammonia concentration on the structural growth of ZnO nanostructures was examined by using several variations of ammonia additions into the reaction, namely from 30 to 300 μL. If we used, for example, 30 μL of ammonia, the final ammonia concentration in the reaction is 36 mM. The experiment was carried out at room temperature.

The sample was then removed and vigorously washed several times using pure water to remove any precipitate on the surface and dried using a flow of nitrogen gas. The sample was also subjected to an annealing process at 350°C in air for 1 h to obtain the effect of annealing treatment on the structures and the morphology.

The morphology of the as-prepared samples was obtained using a field emission scanning electron microscope (FESEM) machine model ZEISS SUPRA 55VP that was operated at an acceleration voltage of 3 kV. The structure and phase purity of the as prepared and the annealed samples were characterised using a Bruker D8 Advance XRD diffractometer with CuK_α _radiation operated at 40 kV and 40 mA. The optical property of ZnO quasi-NRs on FTO surface was characterised using a Perkin Elmer double-beam UV/VIS/NIR spectrophotometer model Lambda 900.

## Results and discussion

**Figure 1:**
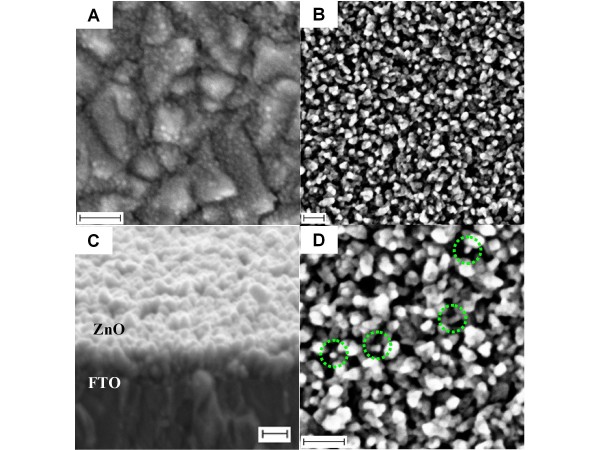
**ZnO nanoseeds on the FTO surface**. **(A) **FESEM image of initial ZnO nanoseeds on the FTO surface and **(B) **after being grown for approximately 5 min in the mixture of 10 mL of 0.01 M Zn(CH_3_COO)_2 _and 36 mM ammonia (standard reaction) producing vertically oriented ZnO quasi-NRs. **(C) **and **(D) **are its cross-section and high magnification images, respectively. Dotted circles in Figure 1D indicate available free-standing individual ZnO quasi-NRs. Scale bar is 100 nm.

We have successfully grown vertically oriented quasi-1D ZnO nanostructures from nanoseed particles on the FTO substrate via a simple and quick growth process, namely 1D crystal growth of nanoseeds via an ammonia-assisted rapid hydrolysis process. In a typical process, the growth took only approximately 3 to 5 min to project spherical nanoseeds into vertically oriented 1D nanostructures. Figure 1A shows a typical FESEM image of initial ZnO nanoseeds that prepared on the FTO surface via an alcoholthermal process. As can be noticed from the image, high-density nanoseeds with a relatively uniform particle size of approximately 5 nm and distributed homogenously throughout the surface were obtained using this approach. The bigger background structures are FTO crystals. After following a growth process in a growth solution that contains, for example, 0.01 M Zn (CH_3_COO)_2 _and 0.036 M NH_3 _(standard reaction), these nanoseeds grew up to large-scale vertically oriented quasi-1D-nanostructures and covered the entirity of the substrate surface (Figure 1B). As revealed in Figure 1B, such high-density quasi-NRs interestingly produce considerably highly porous nanostructured-films of ZnO, a structure that is demanded in photoelectrochemical devices applications for facilitating an active redox reaction. The cross-sectional image taken from the same samples further confirmed that the nanostructures were 1D like structures, which emerge from the initial ZnO nanoseed particles (Figure 1C). The lengths of the structures are approximately 70 nm. However, because of the limited resolution of our SEM machine (Figure 1C), a detailed picture of the vertical orientation of ZnO quasi-NRs that were prepared using this prescription could not be obtained at the moment. Though, a much clearer picture of vertical orientation of ZnO quasi-NRs could be obtained if they were prepared in a higher zinc salt concentration which will be discussed later. As revealed in the higher-magnification FESEM image, the quasi-NRs have the preference to collide and fuse each other at the top-end of the structure, producing big and high contrast particles on the surface. This can be directly related to the result of surface energy minimisation process in ZnO nanocrystals that evolved in such high kinetic activity.

Meanwhile, on the dimension of the quasi-NRs, in spite of such intense aggregates amongst the nanostructures, on the basis of available free-standing individual quasi-NRs (see dotted circles in high-resolution image in Figure 1D); the diameter can be estimated to be approximately 30 nm. It is true that the present quasi-NRs are relatively inferior in terms of morphology and orientation control compared to those currently obtained using other synthetic methods. However, the present technique at least provides an alternative way for a rapid formation of quasi-1D ZnO nanostructures films directly on the surface. Improved and controlled morphology might be achieved later if suitable conditions are obtained, for example via a surfactant modification.

It is important to note here that the nanoseeds are necessary for the preparation of quasi-NRs morphology. If they were absent on the surface, no quasi-NRs products were obtained. Irregular and big nanostructures sometimes were found on the surface instead. However, these could be the precipitates that formed in the solution which then attached onto the surface.

**Figure 2:**
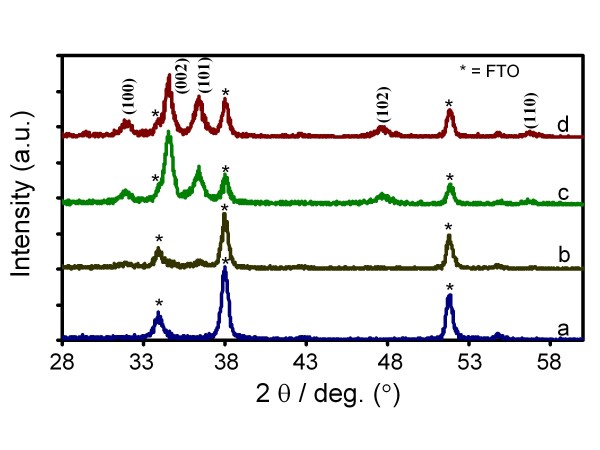
**X-ray diffraction spectra**. X-ray diffraction spectrum of the **(b) **ZnO nanoseeds, **(c) **the as-prepared ZnO quasi-NRs and **(d) **ZnO quasi-NRs after annealed at 350 C. **(a) **is XRD for FTO background substrate.

Unlike in the growth of most metaloxide nanostructures prepared by ammonia [29] or strong base-mediated decomposition such as in the preparation of CuO nanowires [30,31] that produced intermediate metal complexes byproducts [32], the present technique surprisingly produced pure ZnO phase only, evident in the XRD result shown in Figure 2. This definitely could be the result of an effective olation process of Zn-complexes on the ZnO nanoseed surface in the formation of quasi-NRs (will be discussed later) that efficiently transformed them into the pure ZnO. Thus, no Zn-complexes existed in the quasi-NRs structures. The result is particularly important and advantageous because, as for those with the presence of other phases, an after growth annealing process was normally required to facilitate complex removal and produce high-purity ZnO system [29-31]. As can be noticed in Figure 2c, the XRD profile for the as-prepared samples, five prominent peaks at 31.7, 34.4, 36.25, 47.5 and 56.5 besides other peaks indicated by asterisks are apparent on the spectrum. According to the JCPDS (file no. 79-2205), the spectrum can be indexed as the hexagonal wurtzite structure (cell constant of *a *= 3.2501 A and *c *= 5.2071 A) of ZnO with peaks corresponding to (100), (002), (101), (102) and (110) planes, respectively. The peaks with asterisks are assigned to the diffraction peaks from the FTO crystal substrate (see curve a of Figure 2). As also evident in Figure 2c, no peaks related to other zinc complexes are observed, confirming the phase purity of ZnO nanocrystals. A similar spectrum was also obtained for the nanoseeds as shown in curve b, ascertaining the phase purity of the nanoseeds from which the quasi-NRs are grown up. In spite of the fact that the as-prepared quasi-NRs are pure ZnO, we also examined the effect of annealing treatment at 350°C in air on the crystallinity of the samples. However, interestingly the XRD profile was noticed to be relatively unchanged as judged from the height and the width of the peaks, inferring that the as-prepared samples have been through a highly pure ZnO phase so that annealing treatment will give no effect to the modification of their crystallinity. Thus, these results further confirmed the capability of the present technique to produce highly pure ZnO quasi-NRs immediately from the solution.

On the quasi-NRs crystals growth direction, as is evident from the XRD results, the preferred growth orientation of the quasi-NRs might be towards [002] direction judging from the appearance of relatively higher peaks belonging to this crystallographic plane on the spectrum. The peak ratio between this plane and (101) is as high as approximately 1.5 to 2.0, which is much higher compared to the standard ZnO XRD data (JCPDS 01-079-2205), namely approximately 0.5. This result agrees well with those obtained from most ZnO nanorods prepared using, e.g. hydrothermal or other techniques [22,23] in which the [002] is the main crystal growth orientation of the ZnO nanorods. It is true that HRTEM analysis is required for determining the growth orientation of the quasi-NRs. Since the apparatus is unavailable at the moment, a detailed analysis on the crystal growth orientation is being pursued and will be reported in a separate publication.

On the basis of the experimental results, we confirmed that the present approach has successfully promoted the formation of ZnO quasi-NRs from the nanoseed particles. However, at the moment, the mechanism of growth is still not yet well understood. Though, we thought that the growth characteristic of the present system seems identical to the formation of CuO nanowires as reported in [28]. As has been well known, when an aqueous metal salts solution, such as Zn(CH_3_OO)_2 _here, was introduced to the NH_3_, unstable zinc-ammonium complexes might be formed at the first instance. They then rapidly transformed into zinc hydroxides, more stable zinc complexes in solution. In the presence of ZnO nanoseeds on the surface, as confirmed by the XRD shown in Figure 2, these complexes might transform into tetragonal ZnO_4 _phases that initiates the formation of O-Zn-O bridges with the nanoseeds via an olation process [31,33]. Thus, the nanorod structures were projected. In the present work, unsuccessful coordinated zinc hydroxide complexes might apparently be formed, but remained in bulk solution in the form of white-bluish suspension. If attached onto the surface, it can be easily washed out by rinsing with excessive water.

It needs to be noted here that to produce quasi-NRs morphology, the stirring process is necessary in this procedure. If there were no stirring, no quasi-NRs growths were obtained, but a thin films structure composed of quasi-spherical particles instead. It is typical in the present procedure that the zinc complexes were rapidly hydrolysed in the solution upon the addition of ammonia (see growth process in section 2.2.). The hydrolysed complexes easily aggregate on each other forming a bluish colour in solution and at a certain condition they precipitate down to the bottom of the vials. In order to maintain the formation of ZnO quasi-NRs on the surface, the zinc complexes precursors' availability near the nanoseed surface should be sufficient and be controlled. For that reason, the zinc complexes have to be quickly transported to the vicinity of the nanoseed surface by means of stirring shortly after being hydrolysed. Thus, quasi-1D morphology can be formed.

The concentrations of ammonia and zinc salt used in the reaction were found to noticeably affect the structural growth (diameter and length) of the ZnO quasi-NRs on the surface. For the case of the ammonia, firstly, it is noted that the concentration which promotes the formation of quasi-NRs morphology is in the range of 36 to 360 mM. If the ammonia concentration is outside this range, for example lower than this value, no quasi-NRs were obtained, but instead irregular shape particles film formed on the surface. This could probably be associated with the limited precursor availability as a result of a weak hydrolysis process under such low ammonia concentration. Meanwhile, when the ammonia is higher (>360 mM), no or limited quasi-NRs growth was obtained. At this condition, highly compact quasi-spherical nanostructures films were obtained. This could be the result of solution instability under such high ammonia concentration in which the zinc complexes extremely formed and agglomerated in solution that in turn hindered the olation process on the nanoseed surface.

**Figure 3:**
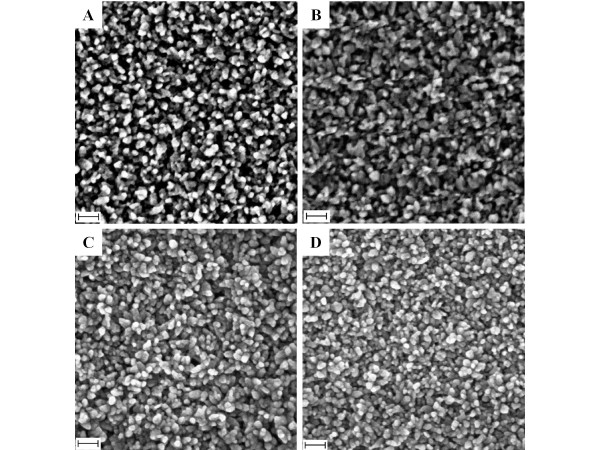
**FESEM and cross-section images of ZnO quasi-NRs**. Prepared in 10 mM of Zn(CH_3_COO)_2 _with different ammonia concentration, namely **(A) **36 (standard reaction), **(B) **180, **(C) **288 and **(D) **360 mM. Scale bar is 100 nm.

Figure 3 shows typical FESEM images of ZnO quasi-NRs that were prepared using four different ammonia concentrations, namely 36 (standard reaction), 180, 288 and 360 mM, with zinc salt fixed at 10 mM. From the image, at a certain ammonia concentration, it is seen that the quasi-NRs efficiently grew up to large-scale producing high-density vertical quasi-NRs array films on the surface. Further analysis on the surface morphology found interestingly that the quasi-NRs density relatively increased with the increasing of ammonia concentration. On the quasi-NRs diameter, to tell the truth, due to extreme aggregation amongst the quasi-NRs, it is quite difficult to obtain the diameter of the quasi-NRs. However, judging from the "grain size" of the nanostructures on the surface that visibly reduced with the increasing of ammonia, it can be remarked that the quasi-NRs diameter should also decrease with the increasing of ammonia. On the basis of available free-standing quasi-NRs, the diameter was seen to decrease from 30 nm to 15 nm for ammonia concentration increasing from 36 to 360 mM, inferring an essential effect of ammonia on the structural growth of ZnO quasi-NRs. Similar to what was obtained in the diameter, the nanorods length was also significantly modified upon variation of ammonia concentration. From the cross-sectional analysis, it was revealed that the quasi-NRs length expanded from 70 to 80 nm when the ammonia concentration was increased from 36 to 360 mM.

In addition, besides modifying the diameter and the length, the variation of ammonia also significantly alters the overall nanorod density on the surface; namely it improves with the increasing of ammonia concentration. Unfortunately, contrary to such enhancement in the density, the augmentation of ammonia induced extreme coalescence amongst the quasi-NRs at their top-end as the result of surface energy minimisation, generating bigger or irregular-shaped nanostructures on the surface that hides the underneath structure of individual quasi-NRs (see Figure 3).

**Figure 4:**
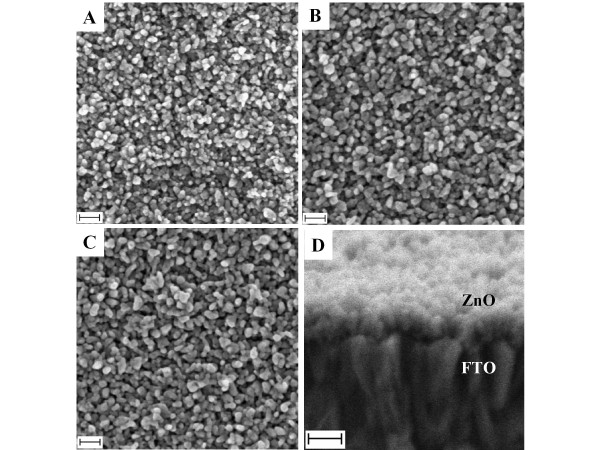
**FESEM and cross-section images of ZnO quasi-NRs**. Prepared in three different Zn(CH_3_COO)_2_, namely **(A) **10, **(B) **20 and **(C) **30 mM with ammonia concentration was fixed at 36 mM. **(D) **is a typical cross-section image for the sample (C). Scale bar is 100 nm.

Similar to what has been obtained in the ammonia concentration variation, a substantial modification on the quasi-NRs morphology was obtained when the zinc salt concentration was altered. In the typical process, the quasi-NRs morphology becomes more rounded and "fatter" with the increasing of zinc salt concentration as can be noticed in the cross-section image in Figure 4D. Analysis on the quasi-NRs diameter found that it significantly increases if the zinc salt concentration was augmented. For example, the quasi-NRs diameter was approximately 30 nm if prepared using the standard solution (zinc salt concentration of 10 mM). It efficiently grew up to approximately 40 nm if the zinc salt used was augmented to 30 mM. As a consequence of the diameter increase, as seen in the image, the quasi-NRs array became denser, producing solid film structures instead of porous morphology as obtained in those prepared using the low zinc concentration. Regarding the quasi-NRs length, it also indicated an effective increase namely from 80 to 110 nm when the zinc salt was changed from 10 to 30 mM, correspondingly, suggesting the controllability of the nanostructure morphology using the present method.

**Figure 5:**
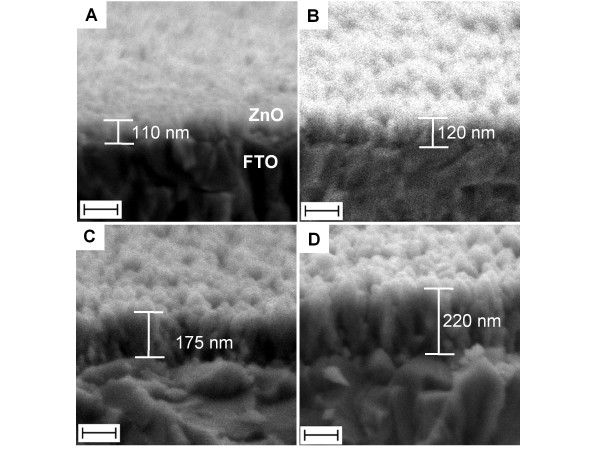
**Cross-section image of ZnO quasi-NRs prepared using different cycle's growth (multiple) process**. **(A) **1, **(B) **2, **(C) **3 and **(D) **4 cycles. The growth solution used contained 10 mM of zinc salts and 36 mM of ammonia. The growth time for each cycle is 4 min. The scale bars are 100 nm.

Up to this stage, the quasi-NRs diameter and density could more or less be adjusted via an ammonia and zinc salt concentration variation. However, frankly, effective control on the quasi-NRs length via one-step growth process was not obtained. We thought that this presumably was correlated with the nature of the reaction in which the zinc salt underwent an extreme rapid hydrolysis and quickly completed in solution, i.e. only within 4 to 5 min of the reaction. Thus, sufficient precursors for maintaining the kinetic growth process are probably unavailable. During the injection of ammonia into the reaction, at the beginning each nanoseed probably quickly projected small nanorod structures with high density on the surface. In an ideal case, the nanorods should further grow until the entire precursors are consumed and promote long nanorod formation on the surface. However, active hydrolysis of zinc salt drove the formation of massive zinc complexes (precursors for quasi-NRs) in solution and aggregated on each other instead of supporting the olation process on the nanoseed surface. Therefore, the quasi-NRs growth was stopped earlier and their length was less developed. However, this could be overcome by using a multiple growth process to provide sufficient precursor materials in order to support a longer quasi-NRs growth. By using a standard growth solution that contained 10 mM of zinc salt and 36 mM of ammonia, the length of the quasi-NRs could be effectively increased from approximately 110 nm (under one cycle growth) to approximately 220 nm if using four cycle's growth process. The results are shown in Figure 5.

**Figure 6:**
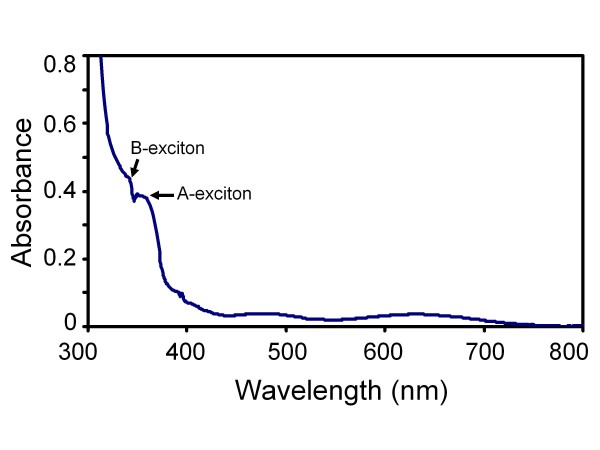
**Typical UV-VIS optical absorption spectrum of ZnO quasi-NRs**. Two separate excitonic characters, namely *A*- and *B*-excitons, were observed in the spectrum, reflecting the ZnO quasi-NRs are high-crystallinity in nature.

Figure 6 shows typical room-temperature optical absorption spectra of the as-prepared ZnO quasi-NRs films. As can be noticed from the figure, one strong and one small shoulder band at the UV region are recognised from the spectrum. These two bands could be associated with two separate excitonic characters of *A*- and *B*-excitons of the ZnO quasi-NRs. The presence of such "clear splitting" in the excitonic bands is quite surprising to us, since this normally only appears in the nanocrystals that contain low defect density; in other words, high-crystallinity [34]. In nanocrystals with low-crystallinity and high defect density, these peaks are broad and will overlap each other forming a single broad absorption band in this region. Therefore, although high-resolution TEM is not available at the moment to confirm the real crystallinity of the nanorods, on the basis of this result it is worthwhile to conclude that the ZnO quasi-NRs prepared using the present approach is high crystallinity in nature. It is true that the *B*-exciton band obtained here is still relatively small. This could be associated with the nature of the quasi-NRs crystallinity, e.g. crystallinity degree or defect content, etc., of the nanocrystals. In addition to these interesting absorption bands, two other bands in the visible region, namely at 450 to 550 nm and 600 to 700 nm, are also apparent in the spectrum. This result is actually different from those normally obtained in most ZnO films, in which no absorption band appeared in this region. Since we used FTO on glass as the substrate, which normally produces an artificial wave pattern at the glass-FTO interface due to an internal reflection, one could have thought that these might come from the contribution of this process to the spectrum. However, since the optical absorption of the sample was recorded via a double-beam spectrometer in which the substrate absorption contribution to the spectrum has been deducted, we conclude that the obtained spectrum could be the special characteristics of the optical absorption of the ZnO sample with the current structure. The bands could be related to several physical processes in the nanocrystals such as singlet excitation in ionised oxygen vacancy [35], zinc interstitial [36-38] or antisite oxygen defect level-related absorption [39]. Even so, a more detailed analysis on the optical properties of the ZnO quasi-NRs on FTO substrate is being pursued and will be reported in a subsequent paper.

## Conclusions

An alternative method for the formation of vertically oriented ZnO quasi-NRs growth on the surface via 1D crystal growth of nanoseeds under a rapid hydrolysis of zinc complexes in the presence of ammonia has been demonstrated. In a typical process, high-density vertically oriented ZnO quasi-NRs with diameter and length in the range of approximately 30 and 110 nm, respectively, was the characteristic of the products. Quasi-NRs were found not to freely stand but leant on each other and combined at the top of the nanarods probably as the results of coalescing process of several quasi-NRs. The growth process was very quick; namely in the range of 4 to 5 min. The quasi-NRs morphology was influenced by the concentration of ammonia used in the reaction. In typical results, the quasi-NRs shape becomes more rounded and fatter with the increasing of ammonia concentration. Meanwhile, the diameter of the quasi-NRs decreased with the increasing of ammonia concentration. The as-prepared quasi-NRs products were pure ZnO phase without the presence of any zinc complexes and feature a relatively high-crystallinity property as confirmed by XRD and optical absorption spectroscopy results, respectively.

As for the mechanism, the quasi-NRs were projected from the nanoseeds probably due to an olation process of zinc complex[31,33], such as zinc hydroxide, on the surface of ZnO nanoseeds, a process that is similar to what has been obtained in CuO nanorods [28].

At present, ZnO quasi-NRs with free-standing and a controlled morphology has not yet been achieved; however, the present method may become a potential alternative for the preparation of ZnO nanorods on the surface. Since the quasi-NRs morphology exhibited a relative dependence on the ammonia and zinc salt concentrations, ZnO quasi-NRs with controlled morphology will be realised if suitable conditions were obtained; for example by utilising the surfactants. The study on this effect is in progress.

## Abbreviations

Quasi-1D, quasi-one-dimensional; quasi-NRs, quasi-nanorods.

## Competing interests

The authors declare that they have no competing interests.

## Authors' contributions

RT carried out nanostructure preparation and characterisation and drafted the manuscript. AAU designed the concept and experiment, analysed the results and revised and finalised the manuscript. MYAR participated in data analysis and ideas. MMS provided the facilities and discussed the results. MO provided the concept of the growth process of the nanostructures. All the authors contributed to the preparation and revision of the manuscript and approved its final version.
